# A literature review exploring how health systems respond to acute shocks in fragile and conflict-affected countries

**DOI:** 10.1186/s13031-022-00484-8

**Published:** 2022-11-15

**Authors:** Kyaw Myat Thu, Sarah Bernays, Seye Abimbola

**Affiliations:** Faculty of Medicine and Health, Sydney School of Public Health, The University of Sydney, Sydney, NSW Australia

**Keywords:** Resilience, Health system, Shock, Acute, Conflict, Fragile, Absorptive, Adaptive, Transformative, Response

## Abstract

Understanding how health systems respond to shocks has become a pressing need to strengthen response efforts. With already fragmented and disrupted health services, fragile and conflict affected [FCA] countries are more vulnerable to shocks. Previous studies have focused more on conceptualizing health system resilience rather than how health systems [especially in FCA countries] respond to or are resilient to acute shocks. To understand how health systems in FCA countries respond to the shocks and what influence their responses, we conducted a review of the literature published between January 2011 and September 2021 on health system responses to acute shocks in FCA countries. We searched Medline, Embase, Scopus, Jester and Google Scholar – 60 empirical studies in FCA countries on response to sudden, extreme, and unanticipated shocks were included in the review. We found that health systems in FCA countries responded to acute shock using absorptive, adaptive, or transformative capacities. These capacities were mediated by four dimensions of context; knowledge, uncertainties, interdependence, and legitimacy. In addition, we identified the cross-cutting role of community involvement [and its self-evolving nature], frontline workers, and leadership capacity. To our knowledge, this is among the first reviews that focus on FCA country health systems responses to acute shock. By highlighting enabling and constraining factors to each type of capacity, this study provides important lessons and practical strategies from FCA countries on how to absorb, adapt and transform in response to acute shocks – thus promoting health system resilience globally.

**Supplementary Information:**

The online version contains supplementary material available at 10.1186/s13031-022-00484-8.

## Introduction

As unforeseen shocks have escalated in recent decades [1, 2] and tested health systems globally, understanding how health systems respond to shocks has become a pressing need. Since the 2014–2016 Ebola outbreak in West Africa, the concept of resilience has received much attention among health system researchers and policymakers [2, 3]. Before that, ‘resilience’ had been widely used in other disciplines. In the physical sciences, resilience is considered the capacity of a system to return to its original state after a disturbance [2, 4, 5]. In the ecological sciences, it means the absorptive capacity of an ecosystem during shock [2, 6, 7]. In the social sciences, it is recognized from the perspective of complex adaptive systems [2, 8, 9]. As Blanchet et al. note, the definition of resilience remains contested and still ambiguous for developing consistent strategies [1, 10–14]. However, resilience is widely acknowledged as an attribute or ability necessary for health systems to respond to disturbances, including acute shocks [15–18].

In relation to resilience, the term ‘acute shock’ means a sudden and generally negative event, which has substantial impacts on the livelihoods of people and the function of systems [19, 20]. The narrower and more precise definition of an acute shock is a sudden and often surprising event that causes an additional burden to the health system, most often for a short period [1]. Health system shocks rarely act in isolation. They often occur in the context of long-running stress to systems. However, response to acute shocks has become an important focus of health system research because of growing occurrence of sudden and extreme disturbances, such as epidemics and pandemics [19], compared to slowly occurring chronic health system strains, such as long-term underfunding, population ageing or long-term political instability [1]. This review will focus on health system responses to acute shocks rather the chronic stresses. Health systems need to provide undisrupted basic health services, protect people and prevent another shock that may result from poor management of recent or current ones [1].

The recent and ongoing COVID-19 pandemic underscored the complexity of health systems in fostering resilience to shocks. During acute shocks, countries need strong, robust health systems to respond to increasing demand and deliver essential health services [21]. The ability of health systems – which has been defined in relation to resilience as the ability to absorb or adapt, or transform – to provide essential health care [1, 11, 14, 22–24] varies significantly in the face of shocks [1, 25]. While stronger health systems more successfully limited transmission and deaths from COVID-19, fragmented and weak health systems have struggled to cope with the accelerating number of cases [1].

Currently, billions of the world’s population reside in fragile and conflict-affected [FCA] countries with unstable political and economic situations, and with fragmented and disrupted health care services. Two-thirds of the world’s population living in extreme poverty are in FCA countries [26, 27] which are increasingly vulnerable to shocks [2, 28]. World Health Organization [WHO] defined FCA states as “a group of countries or territories categorised by the World Bank’s Fragile, Conflict and Violence group based on their financial and security status, with an updated list being released annually from 2006 onwards” [WHO P1] [29]. FCA countries encounter enormous burdens because of the destruction of health care infrastructure, loss of property, and massive displacement of populations [21, 30, 31]. Nevertheless, some countries, such as Guinea, Liberia and Sierra Leone, have demonstrated a greater ability to withstand shocks [32]. Hence the need to understand the differences and similarities among responses in FCA countries to shocks.

However, studies on health system resilience in response to acute shocks are rarely conducted in FCA countries [33]. While health system resilience is well explored conceptually, there is still limited evidence and insight on what makes health systems, especially in FCA countries, respond to acute shocks and generate resilience [2]. As indicated, the challenges and vulnerability to shock are context-specific, such that single descriptions and universal characterizations of responses to shocks are problematic [28, 34–36]. This review conceptualises resilience as health systems’ capacity to absorb, adapt, and transform when exposed to a shock [Blanchet et al. P431] [23, 37–39], and in line with WHO’s definition of resilience as “the ability to prepare for, manage [absorb, adapt and transform] and learn from shocks” [WHO P6] [1]. In this review, we aim to answer the question: how and under what circumstances do health systems in FCA countries absorb, adapt, and transform in the time of acute shocks?

## Methods

We conducted a literature review that included quantitative and qualitative studies to explore how health systems in FCA countries respond to acute shocks by exploring the links between the response, its enabling or constraining factors, and the capacities that explain the relationship.

### Search strategy

A literature search was conducted in September 2021. We used different databases; Medline and Embase via Ovid and Scopus database, JStor and Google Scholar [from 2011 to September 29, 2021] using the following terms: #1[Health system adj2 [respon* OR behaviour*]].tw AND #2[[shock adj2 [acute OR health system]] OR [Outbreak* OR Pandemic] OR [Disaster OR Earthquake OR Flood*] OR [Armed conflict OR War OR Crisis] OR [Global health security]].tw. The search terms were adapted from previous studies conducted on health system resilience, health system response and health system shocks [23, 24, 34, 40]. Shock-related search terms were included to cover the broad range of health system shocks ranging from natural to man-made. The search terms were discussed among reviewers interactively and tested in Google Scholar until we achieved consensus.

We included English language articles published between 1 January 2011 and 29 September 2021. We included studies done in any FCA country listed in the World Bank Group [WBG]’s fragile and conflicted-affected situations from the year 2011 to 2021. We included empirical studies that focus on sudden, extreme and unanticipated external disturbances or challenges to the health system. Since health system response to acute shocks is expected to be time and context-specific, papers with comparative analysis across geographical locations/settings and across time/periods were included. Only empirical research that observed or measured the events and derived insight from actual experiences to shocks were included rather than theoretical or conceptual papers. We excluded studies unrelated to the impact of acute shock events on health systems, including any health system shocks that occurred as a direct result of existing risks or stressors.

### Data extraction and categorization

Application of the search terms with initial filtering on FCA counties yielded 115 entries from Medline and an additional 1124 entries from Scopus. The Medline result was manually filtered and reduced to 17 articles by excluding articles from high-income countries. The search yielded additional 13 entries from JStor and 70 from Google Scholar. After entries were subsequently merged and duplicate excluded, the total number was reduced to 1221 publications.

We eliminated non-relevant articles by title, abstract and full-text screening. Items were excluded by title and abstract screening if they were not empirical papers [e.g., commentary, editorial or systemic review] or were not concerned with the health system experience [e.g., human immune system response to disease outbreaks] or studies focused on chronic health system stressor [e.g., health worker shortage], or preparation for anticipated shocks [e.g., simulation for anticipating shock]. We also excluded articles that were concerned with system shocks but did not focus on the health system [e.g., shock impact on agri-food system].

**Fig. 1:**
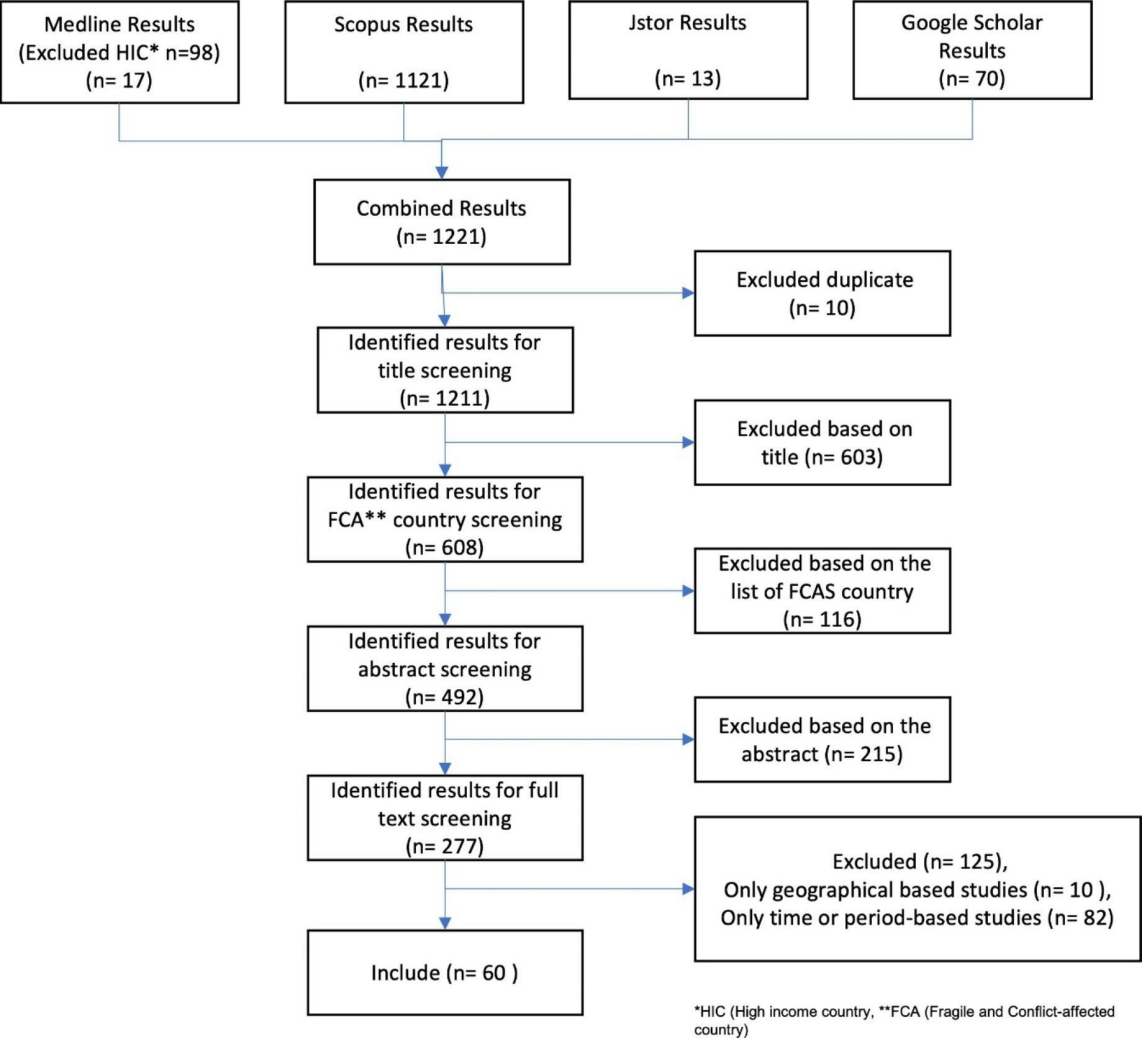
Screening process of papers obtained through searches

After abstract screening, 277 articles remained of which 217 references were excluded after full-text screening (see Fig. 1). Abstract and full-text screening were carried out based on the inclusion and exclusion criteria, the reviewers’ judgement, and joint discussion among reviewers for clarification or achieving consensus along the process of interpretation of findings and writing [KMT in consultation with SA and SB]. A total of 60 studies were finally included in the review (Appendix 1) Appraisal of the contribution of any section of data [within a document] was based on two criteria: “relevanc*e* – whether it can contribute to theory building and/or testing; and rigor – whether the method used to generate that piece of data is credible and trustworthy”[41]. Relevance of the selected articles was assessed against the inclusion and exclusion criteria and relevance of the extracted data was assessed through extensive discussion among authors against the conceptual framework of Blanchet et al. [23]. To assess rigour the authors ensured the fragments of data extracted from each study was reliable based on the design and on triangulation from a range of other studies included in the review. The findings were interpreted and constructed through frequent discussion among authors to minimize bias, and the coding of the data and analysis process was done based on the conceptual framework of Blanchet et al. [23].

Data extraction was carried out using Microsoft Excel. We extracted data regarding general study information, study question, design, the unit of analysis, and study design. Into Excel spreadsheets, we entered the health system response of interest in each study, including verbatim extractions of text relevant to understand the links between the response, its enabling or constraining factors, and the ‘capacities’ that explain the relationship. Data and insights were extracted from various parts of articles: from introduction, description of methods, reported results, and interpretative reflections or discussion sections. When they were not immediately apparent from the introduction or findings of a study, we relied on the interpretation and explanation of the authors on the different categories of data that we extracted from each paper. Data extraction was guided by the conceptual framework of Blanchet et al. [23] as it covers several concepts highlighted in previous resilience studies.

Data were extracted using the following definitions of capacities [Blanchet et al., P 432][23].

- “Absorptive capacity relates to the capacity of a health system to continue to deliver the same level [quantity, quality and equity] of basic healthcare services and protection to populations despite the shock using the same level of resources and capacities”.
- “Adaptive capacity is the capacity of the health system actors to deliver the same level of healthcare services with fewer and/ or different resources, which requires making organisational adaptations”.
- “Transformative capacity describes the ability of health system actors to transform the functions and structure of the health system to respond to a changing environment”.

Data were extracted using the following categories of interlinking ‘contextual’ dimensions across capacities [Blanchet et al., P 432][23].:

- Knowledge: “capacity to collect, integrate and analyse different forms of knowledge and information”;
- Uncertainties: “ability to anticipate and cope with uncertainties and surprises”;
- Interdependence: “capacity to engage effectively with and handle multiple- and cross-scale dynamics and feedbacks”; and.
- Legitimacy: “capacity to build or develop legitimate institutions that are socially accepted and contextually adapted”.

We searched passages of each paper for system response to shocks – and these were coded as ‘outcomes’. To identify [failed] resilience as an outcome, we actively looked for what capacity made health system produce the outcome: absorptive, adaptive or transformative. Each capacity was accompanied with notes about factors that either enabled or constrained them—and these were labelled as context. As health systems are embedded within politically, socially and economically complex structures and linked across scale, we identified, in terms of context, how well responses to shocks combine and integrate different forms of knowledge into action [Knowledge], deal with emerging uncertain situations [Uncertainties], engage and coordinate within the complex structures and across scale [Interdependence], and establish the trust and leverage the community ownership [Legitimacy] to understand the enabling and constraining circumstances. We further identified other features of dynamic interaction of health systems that may also prevent or lead to system capacity for resilience.

## Findings

Across the selected papers, health systems in FCA countries encountered different types of acute shock ranging from natural to man-made disasters. Disease outbreaks, from rarely occurring events like meningococcal disease outbreak to large scale events like the Ebola epidemic and COVID-19 pandemic were the most type of acute shocks [42 out of 60 selected articles], followed by 12 articles on war or conflict, and then 4 disaster-related studies. A few studies, 4 out of 60 studies focused on different types of shocks, categorised as multiple (Table 1). Even though the types of shocks are different with varying levels of intensity, they have similarities in their sudden and often surprising impact on the already fragile health systems.

**Table 1:** Types of shocks identified in the studies

Type of shocks	Number
Outbreak	42
Disaster	4
War or Conflict	10
Multiple	4
Total	60

Of the 60 studies, 22 discussed whole health system response to acute shock. Most studies focused on one health system function: 6 studies on governance, 20 studies on service delivery, 5 studies on human resources and 3 studies on information systems. Although studies that focus solely on medicines and technologies and finance were not among those included in our analysis, these functions were covered in studies related to the whole system. The remaining 4 studies are related to community or peoples’ response in time of acute shocks. Although health system researchers often focus on one system function at a time, our review found that all the health system actors interact to generate a particular type of response to acute shocks.

In our analysis, we identified three health system capacities that interplayed in health system response to different types of acute shocks: ‘Absorptive capacity, ‘Adaptive capacity and ‘Transformative capacity’. We used superscripts to refer to which of our findings are linked to the 60 publications included in the review - which were presented as a second list of references [see Supplementary Appendix 1]. Because of the different settings and types of acute shocks, the capacity we identified represents tendencies of health system response to acute shocks in FCA countries but does not represent any countries specifically, but the immediate setting of a response. Our analysis focused on capacity and dynamic responses concerning acute shock rather than specific countries and shocks.

## Absorptive capacity

Of the 60 studies conducted across 69 different settings, responses in 60 settings showed varying levels of absorptive capacity, and 9 did not exhibit absorptive capacity.

### Knowledge

Following acute shocks, most of the health systems in FCA countries with studies included in our review used existing capacities and resources of system actors to limit the impact of the shock. At the initial phase of acute shock, leadership, either institutional or political, is central to translating the existing knowledge and resources into action ^2,10,17,23,25,28,33,42,60^, improving collaboration among system actors, promoting community engagement and attracting donor revenue. Notably, in our study, the term leadership refers to either institutional leadership – i.e., the capacity of multiple organizational actors to engage in information sharing and collaboration for emergency preparedness and response [42, 43]; or to political leadership – i.e., the capacity of political and bureaucratic leaders to make administrative decisions [42, 44] essential for crisis management like acute shock events in the health system [42, 45]. Integration of knowledge starts mostly top-down to provide policy guidance on implementation and resource allocation^17,23,25,28,33,42,60^. Systems’ ability depends on local or subnational leadership capacity to recognise and respond as necessary before reporting^2,10,30^. Settings with the ability to combine and integrate different forms of knowledge appeared to demonstrate greater absorptive response^2,3,5,9,10,11,46,60^compared to those with limited ability^8,9,10,13,17,20,22,26^. Some settings with weak institutional or political leadership [e.g. centralized decision making, lack of collaboration with other actors, and delay in the administrative decision] were unable to limit the immediate impact of shocks and initiate a rapid response^8,9,10,13,16,17,20,21,22^.

Health systems’ existing capacity and governance for managing knowledge are linked to prior experience of similar events or anticipatory planning. Systems^9–11,37^or health workers^57,58^ with prior experience of managing a similar shock found such experience valuable in taking prompt response. However, settings with no prior experience^9,10,56^ or that failed to learn from the past shocks^20,41^ have less structural readiness, leading to delayed response. Moreover, settings that have anticipated the shock and have functional surveillance systems had the ability to identify the shock and support decision making to initiate a rapid response^5,23,46,60^. The stronger the surveillance system in place, the better the decision making and resource allocation to mitigate the impact of the shock. Settings with disrupted or lack of surveillance systems failed to detect, report, and initiate response to shocks^13,16,17,20,41,45,47,50^.

### Uncertainties

Health systems in FCA settings faced uncertainties in prioritising action, allocating resources, and managing acute shocks. Uncertainties required leadership in using knowledge to foster dynamic interaction and networking among decision-makers, frontline workers and community members so that actions reflect the needs of the community in real-time to absorb shocks and initiate rapid response^2–5,9−11,17,23,25,28–30,33,35,42,52,57,60^. Health systems with good community engagement and skilful and motivated front-line health workers performed well in dealing with uncertainties ^1,2,3,11,29,30^. Settings with options to provide financial support flexibly and ability to supply necessary equipment and train as necessary are more absorptive as they are better at providing timely and targeted action to the shocks^1,2,11,17,29,35,46^. When there was disruption of services provision, settings that supported community mobilization through training, providing allowance and supplies ensured the continuity of services at the community level^29,30,33,36, 58^. However, weak leadership compounded by lack of funding and inadequate supplies impaired the active involvement of frontline health workers and community members in the immediate responses^2,7,8,16,17,20,21^. Inadequate staff, training, necessary equipment and infrastructure also challenged the ability of health systems to deal with uncertain situations^7,8,1016,22^.

### Interdependence

Some settings exhibited the ability to coordinate and communicate across levels of the systems to absorb and recover from acute shocks. Effective leadership created national unity and improved inter and intra-governmental coordination^1,3,5,10,11,17,23,25,29,60^. Multi-sectoral, multi-level and multi-stakeholder involvement contributed to better coordination and implementation across levels^2,3,5,10,11,15,27,29,52^. Strong relationships between community and government improved community ownership in addressing the impact of shock^30,35,58^. On the other hand, settings with a highly politicised response [e.g. where political parties took actions to advance their immediate or partisan political interest], and heavy involvement of INGOs and donor agencies were less absorptive because of power imbalances in decision-making^1,9,17,21,41^especially in settings with donor dependency, lack of decentralization and weak leadership capacity. However, the prominence of INGOs can be a manifestation of weak state administration and/or dysfunctional governance. Centralization lessened the opportunities for stakeholders to exchange ideas and delayed decision-making that are crucial for absorptive capacity ^9,16,22,2631^. Poor relationship among the community and public health sectors including lack of inter and intra-sectoral coordination were also barriers in the continuation of basic health services^17,20^.

### Legitimacy

Governments conducted regular consultation with stakeholders through assessment or advocacy enabled their ability to provide a consistent action plan in times of shock^1,2,23^. Active involvement of the community in the consultation process and engaging them in the national response built trust between the community and government, and improved local ownership^29,35,36^. Different communication strategies were used in different settings: social networks, religious structure, media, campaigns, to reduce misinformation or malpractice in the community^2,5,11,17^. However, settings with deep-rooted mistrust between communities and governments could not implement a people-centred response^2,8,9,17,21^. Delay or inappropriate action taken by governments also hampered community engagement in the response^21,31,32,56^.

## Adaptive capacity

Among 69 settings, 38 had responses with varying levels of adaptive capacity and 31 responses did not exhibit adaptive capacity. Of the 31 responses without adaptive capacity, 17 exhibited absorptive capacity when the shock hit. In responses with adaptive capacity, 29 had both absorptive and adaptive capacity, but 9 exhibited only adaptive capacity. Adaptive responses occurred more in settings that had initially exhibited absorptive capacity.

### Knowledge

Settings with the ability to establish new data collection and information management systems with the changing situation of acute shocks performed well compared to others^2,3,6,19,21,25,26,40,60^. Governments used Monitoring and Evaluation systems or new strategies to ensure that their response reflect the realities and to understand the effectiveness of the response in real time^2,3,6,26^. Some settings’ capacity to collect information through rapid assessment or community feedback enabled their ability to adapt with subsequent efforts even though they could not exhibit absorptive capacity in the early stages of their response^19,40^. One setting, which initially had exhibited absorptive capacity, lost ability to adapt to rapidly changing situations because of poor identification and data collection ^17^. Adaptive capacity also depends on leadership to process knowledge into action in timely manner – to establish response teams, and to develop capacity-building plans and implementation measures^1,2.5,12,14,22,33^. Leaders who were aware of the importance of community and frontline health workers were better at adapting and managing to build trust with the communities at the time of shocks^21,24,32,41,50,51,53,54,56^ .

### Uncertainties

Health systems’ adaptability to uncertain situations largely depends on actors’ interaction and partnerships to develop new strategies to support systems across scales and levels^1,3,4,6,24^. Partnering with international or national stakeholders enabled the continuation of basic health services by obtaining funding, supplies, or technical support^1,3,4,6,24^. With government leadership, consultation or brainstorming sessions were held at each level to ensure that the response was comprehensive and reflective of end-users’ needs^12,14,39^. Integration of activities [e.g., integrated TB/HIV/COVID-19 testing, integrated local food production and nutrition response] improved health system ability to limit the impact of shocks^9,27^. Although settings with absorptive capacity had greater government capacity to adapt to uncertainties during shocks^1,10,3,7,1012,15,17,24^, community and frontline health workers played a major role in adaptive response in settings with limited absorptive capacity^2,12,19,23–25,27,31,32^. Adequate health system supplies enabled heath care networks and expanded their capacities^12, 25,27,31^. Community empowerment and engagement provided adaptive capacity to counter shocks and limit the collateral impact^2,12,19,32^. In the face of service gaps during shocks, communities empowered themselves and served as a natural platform to undertake activities and support the health system indirectly ^2,25,19,32^.

### Interdependence

We identified the complex nature of health system actors interaction across scales in settings with adaptive capacity. Involvement of multiple sectors such as health/non-health sectors, local/international organizations at local and national levels^2,3,9,11,14,25^ influenced health system’s adaptive capacity to respond to the shock^2,6,21,26,41^. Different resources were used across settings, e.g. health workers were safeguarded by the military or police or supported technically or financially by INGOs for adapting emerging situations^2,4,9,25,54^. Lack of collaborative stakeholder effort diminished adaptive capacity^5,17^. Local or national leaders with ability to engage social brokers in the response performed better in exploring community concerns and adapting the actions as necessary ^10,18,24,52^. In some settings, informal networks of healthcare providers [either through deliberate policy action or through spontaneous grassroots action] managed to limit the health services gap during shocks^19,24,27,51^.

### Legitimacy

Community engagement in response to shocks reduced the risk of trust issues and enhanced health systems’ ability to adapt to changing situations^6,10,11,26,57^. Some settings improved community participation by conducting cultural analysis or using innovative risk communication strategies^10,11^. Some responses built trust between health officials and communities by sharing information transparently through media, social brokers, and in-person discussion^2,19,24^. Installation of community-based programs favoured community to engage in the response and built trust^11,14,27,32,50^. Building capacity at health centres, but also community consultation and information exchange between community and health staff improved the legitimacy of the response^6,24,53,56^.

## Transformative capacity

In the 69 settings, 15 responses showed transformative capacity, while the remaining 54 did not. Among responses with transformative capacity, 8 showed all three capacities [absorptive, adaptive, and transformative]; 6 showed both adaptive and transformative capacities, and 1 showed only transformative capacity.

### Knowledge

Some FCA settings transformed their functions and structures to respond to shocks. Ability to coordinate stakeholders across scales facilitated the translation of knowledge into transformative action^24,41,59^. Settings with such ability used different approaches to collect information, coordinate with stakeholders, and deliver contextualized strategies^2,11,24,25,41,50,59^. Government ability to use information from surveillance systems, community feedback mechanisms or national or international experiences, to identify resources and gaps influenced their transformative capacity^6,15,59^. Systems with ability to engage the community through coordination and monitoring facilitated community to engage in transformative action as necessary for recovery^6,24,41,50,53,59^.

### Uncertainties

For already fragmented and weakened health systems in FCA settings, the ability to transform may require strong leadership to process information, plan, react and transform as necessary at different levels^15,60^. Since different systems have different levels of capacities and needs, some responses incorporated consultation and advocacy with stakeholders and used innovative tools to understand the levels, needs and monitor their response ^15,24^. In some settings, changing practices, such as from centralized care to home-based care or restriction of practice in the time of disease outbreaks, enabled responses to limit the impact of shock^6,11,,24^. Settings that exhibited absorptive and adaptive capacity were better at dealing with uncertainties^2,3,9,11,25,58,60^.

### Interdependence

Settings that have the ability to engage governmental or non-governmental agencies other than in the health sector exhibited the transformative capacity to make a change in the system^3,6,11,15^. The role of I/NGOs, local councils or communities as social brokers and innovative communication channels enabled the quality and compliance of transformative process^2,3,24,59,60^ – as did self-evolving grassroots community action in transforming their own practices in response to the emerging situation^2,3,25^.

### Legitimacy

Settings with transformative capacity adopted community engagement and inclusive approach to decision-making, which improved trust between communities and governments^2,6,11,25^. Some responses improved the accountability to communities by facilitating more stakeholder involvement in the response^2,3,11,24,58^. Empowering and including communities in responses improved community acceptance of transformative changes and response^2,9,25^.

## Discussion

When shocks hit, health systems in FCA countries respond in different ways using either one or all capacities [absorptive, adaptive, and transformative] to protect their people. Acute shocks of any type, such as a pandemic, disaster, or conflict, come externally and put pressure on health systems [46]. Whether they succeeded or failed in their response, almost all of them used absorptive capacity initially. For instance, when COVID-19 took the world by surprise, FCA countries, such as Arab Levant Countries [ALCs], struggled to initiate responses due to suboptimal infrastructure compounded by political unrest^17^. All ALCs [i.e. Palestine, Lebanon, Syria, Iraq, and Jordan] initiated responses using existing resources to absorb the shock. However, only Jordan and Lebanon managed in terms of case detection, reporting and mortality in COVID-19 response^17^ given their existing government capacity and strong relationship with communities favoured immediate response to absorb the shock of COVID-19^17^. The failure of governments to take immediate or appropriate measures has been attributed to political influences of conflict in the countries with the lowest political stability index, such as Palestine, Syria, and Iraq. The findings of our study are in line with the findings of ecological resilience studies on absorptive capacity, which is the initial ability to stabilize the system within the same state over a range of disturbances and management actions [6, 47, 48].

However, some FCA countries demonstrated the capacity to adapt or transform rapidly as necessary to limit acute shock impact even under limited resources^12,18,19,40^. When cyclones Idai and Kenneth hit Mozambique, lack of information on geographical accessibility to health care was the main issue in initiating a response^40^. Mozambique adapted post-disaster accessibility modelling to identify service coverage and geographical constraints for planning, allocating resources and recovery. In the other scenario, although Boko Haram insurgency disrupted health services in 2011, parts of Northern Nigeria exhibited transformative capacity for accessing quality maternal, newborn and child health services^15^. They reformed their health systems to improve government accountability and resource management in health services provision^15^. Health systems in FCA countries can have different responses and different levels of resilience based on their capacity to absorb, adapt, or transform in the face of shocks, as conceptualised by Blanchet et al., [23]. In this regard, as Topp argued, health system resilience refers to the ability rather than an outcome [23].

Among FCA countries that encountered extreme crises, some performed better by taking action rapidly in changing situations^2,3,9,11,25,47,58,60^. They exhibited resilience by resuming systems function [absorptive capacity] and adapting as necessary with the changing situation even in a time of limited resources [adaptive capacity] and transforming new systems [transformative capacity]. Notably, absorptive capacity, which Abimbola and Topp conceptualized as robustness, contributes to systems’ capacity to adapt and transform [11]. Already weak health systems compounded by socioeconomic and political instability are vulnerable in the face of shocks^1,4,8,17,20,21,24^. Health systems without robustness are less likely to be resilient. However, adaptation happened in some countries [Somalia, Chad, Cameron, Niger and Yemen] without an existing absorptive capacity^6,59^. Their coordination ability, information management skills and adaptability made them different from others^6,59^. These settings exhibited adaptability without robustness, which Abimbola and Topp described as coping rather than resilience [11]. As they are humanitarian settings with protracted conflicts, they may have anticipated the acute shock events. Although such settings have the risk of overoptimization to well-known shocks, we did not identify such risks in this study^6,59^. Likewise, adaptation under duress may show ‘mal-adaptation to undesirable states’ [14], which allows the system to limp along but can become entrenched, such that the undesirable aspects of the adaptation are difficult to correct after the shock.

In this study, we identified factors that make systems respond in different ways (Table 2). Across the findings, the roles that leadership, front line workers and community played were cross-cutting, and responses varied with the strengths and weaknesses of those roles. In settings with absorptive capacity, leadership was less influential than other capacities. Rather, response mostly depended on how well health systems prepared in advance, similar experiences to the shocks in the past and the existence of strong bonds between community members and the government. In addition, having competent and motivated front-line health workers and community involvement made some countries different to others in their ability to mount quick responses to emerging situations. Settings lacking in those abilities exhibited limited absorptive capacity. Leadership for knowledge management and engaging communities in the response enabled adaptive response to changes and effective use of resources for providing basic health services. The activities of community and frontline workers – including spontaneous grassroots responses – influenced transformative capacity. However, the ability and willingness of governments to receive community and stakeholders’ feedback were essential for transformation.

**Table 2:** Enabling factors for absorptive, adaptive, and transformative capacities

	Enabling factors
**Absorptive capacity**	• Regular consultation of governments with different levels of stakeholders • Previous experience or anticipation of shocks • Competent and motivated front-line health workers • Community involvement • Existence of strong bonds and kinship between community members and the government • Strong leadership capacity • Limited involvement of INGO and donor agencies • Depoliticised and decentralised decision-making • Strong surveillance system • Financial and equipment support to frontline health workers • Trust among the community and public health sectors • People-centred approach
**Adaptive capacity**	• Leadership adaptability to complex situations • Knowing the important role of CHW and frontline health workers • Integration of activities • Ability to establish new data collection and information management systems • Empowering and engaging community • Ability to identify and use social brokers • Involving informal networks of healthcare providers • Community engagement and inclusion • Engagement of government in the response • Community’ collaborative and self-evolving capacity • Strength of the surveillance system
**Transformative capacity**	• Stakeholder consultation and advocacy • Learning through community feedback mechanisms • Using innovative tools for monitoring

**Fig. 2:**
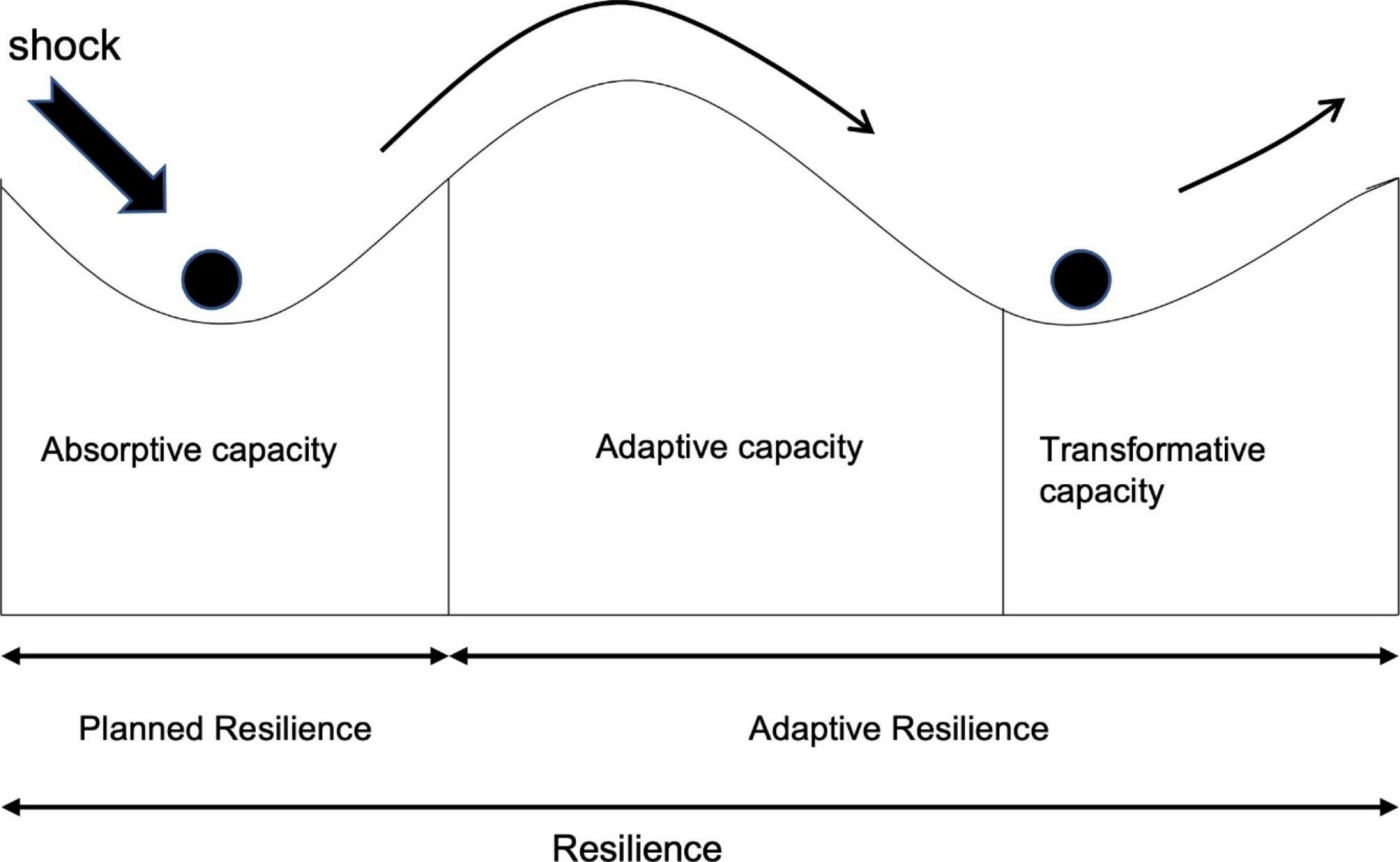
Interlinking nature of health system capacities with two dimensions of organizational resilience. [adapted from ecological resilience and adaptive capacity [48]]

We identified links between the capacities and the two dimensions of organizational resilience – planned and adaptive resilience – which Walker et al. conceptualized as core components of resilience [2, 49], both interconnected to create organizational resilience (Fig. 2). Findings in this study suggest that health systems in FCA countries can have just one or both dimensions of resilience in response to acute shocks. When systems encounter shocks, they exhibit absorptive capacity as a first-order response to limit vulnerability. Despite the fragility and fragmented state of health systems in FCA countries, many used existing capacities and resources to mitigate the negative consequences of shocks^2,5,9,10,17,23,25,28,30,33,35,42,58,60^. The use of those existing capacities and resources , which is absorptive capacity, to minimize the effect of shocks has been discussed in the organizational resilience literature as a first-order capacity [2, 19]. Our findings on the ability to absorb or recover at the early phase of shock are linked with the organizational resilience concept of planned resilience. We observed that this planned resilience may occur independently of adaptive resilience

The interactive nature of complex adaptive systems meant that the three capacities interacted as health systems responded to acute shocks. As described in ecological and health system resilience studies, settings with absorptive capacity are better able to adapt or transform^1,2,3,4,9,10,11,25,27,48,51,57,58^. For instance, Uganda’s ability to absorb shocks in response to an Ebola outbreak influenced system adaptability to changes. Developing a new surveillance team and supporting mechanism improved service accessibility^9^ and enabled trust when implementing and transforming communication strategies and burial practices in Uganda. The adaptive and transformative capacities, mentioned in organizational resilience as adaptive resilience [2, 49], interact with planned resilience to limit the impact of shocks and prepare for recovery.

However, the three capacities that framed our analysis interact also in a non-linear fashion. Some countries, such as Myanmar and Palestine, could not adapt to the emerging situation during the shock, even though they expressed their ability to absorb the shock initially^5,17^. They handled the shock initially with pre-existing leadership capability and information available from surveillance systems, but inadequate infrastructure and financial support hampered their adaptability to increasing demands during shocks. This indicates that adaptive and transformative capacities are more influential in dealing with uncertainties and emerging situations during shocks. It aligns with Walker et al.’s argument that adaptive resilience is more influential than planned resilience in the context of uncertainty in responding to acute shocks [49].

In the face of shock, stabilizing the system is challenging for already fragmented and weakened health systems. Not all FCA countries have favourable conditions or capacities to absorb, adapt, or transform in response to shocks. Their capacity depends on context – but leadership stood out especially as important for information management, resource allocation, planning and initiating effective response^2,3,7,10,46,55^ in uncertain situations^2,4,6,9,12,14,24,31,39^. The ability to manage knowledge and deal with uncertainties enabled trust between governments and communities, and encouraged community and stakeholders involvement in the responses^1,3,10,11,14,18,19,^. Weak leadership in translating knowledge into action and centralized decision-making hampered inter or intra-sector collaboration across levels^1,2,5,9,20,26,31^. Heavy INGO and donor involvement led to power imbalances that hampered decision making^1,2,9,11,16,31^, transparency and relationship between governments and communities. However, in some countries, the collaboration, initiative, and self-evolving capacity of communities ensured service continuity and government accountability^2,3,19,25,29,30,31,33,50^. This indicates the need to further explore community resilience and self-evolution to fill gaps in accessibility and continuation of services during shocks in FCA countries.

Overall, to our knowledge, this is the among the first review focuses on health system responses to acute shock in FCA countries. Our exploration provides substantial contributions to the health systems literature by analysing the complex, adaptive and iterative nature of health systems in their response to acute shocks. The inclusion of a broad range of articles from multiple countries through our broad inclusion criteria and systematic search ensures that our findings are comprehensive. Our review adds to previous efforts to conceptualise resilience: absorptive, adaptive and transformative by Blanchet et al. [23] and the conceptualization of resilience by Abimbola and Topp as adaptability with robustness, such that adaptability without robustness is coping [11]. Using the framework developed by Blanchet et al. will allow others to add to our work in a way that further consolidates the literature.

While we attempted to apply a framework for assessing the health system response, our analysis involves the judgement of reviewers that may potentially bias the findings. Another potential limitation of this study is that we included different outbreaks-related articles in the review, especially COVID-19 related articles. Response to COVID-19 continues to evolve, and so there is a possibility of the bias of including only reports of early response to the pandemic. However, the framework we used in this review guided us to assess system capacities in response to any type of shock that have similarities in their sudden and often surprising impact on already fragile health systems. Another potential limitation is that our study focuses only on acute shocks, which are sudden and extreme, but health systems in FCA countries are also experiencing chronic shocks [stresses], which put a burden on the health systems. Future studies focusing on health system response to chronic shock [stress] in FCA countries will be valuable.

## Conclusion

We reviewed the literature on health system response to acute shock in FCA countries from 1 January 2011 to 29 September 2021. We included 60 empirical studies of responses to sudden, extreme, and unanticipated external disturbances or challenges in FCA countries. We identified that health systems in FCA countries can respond to the acute shock in different ways using one, two or all three systems capacities [absorptive, adaptive, and transformative capacities] mediated by context along four dimensions: knowledge, uncertainties, interdependence, and legitimacy. Findings from this study provide important lessons from and for FCA countries in their response to acute shocks, which are not limited to their experiences, and practical strategies to absorb, adapt and transform in relation to shocks. The findings also highlight enabling and constraining factors that health systems in FCA countries may address. The role of leadership, the competence of frontline workers and community engagement were identified as important in systems’ ability to continue basic health services in the face of shocks. Findings from this study suggest that future research should focus on community resilience to acute shocks in FCA countries and studies that explore health system responses to chronic shock [stress] in FCA countries.

## Electronic supplementary material

Below is the link to the electronic supplementary material.

Supplementary Material 1

## Data Availability

All data generated or analysed during this study are included in this published article [and its supplementary information files].

## Abbreviations

ALCs: Arab Levant Countries.
COVID-19: Coronavirus disease of 2019.
FCA: Fragile and conflicted.
HIC: High income country.
HIV: Human Immunodeficiency Virus.
INGOs: International Non-governmental Organizations.
NGOs: Non-government Organizations.
TB: Tuberculosis.
WHO: World Health Organization.
WBG: World Bank Group.
