# Supplementary material for: A literature review exploring how health systems respond to acute shocks in fragile and conflict-affected countries
Source: Confl Health. 2022 Nov 15;16:60. doi: 10.1186/s13031-022-00484-8 (PMC9665009; doi:10.1186/s13031-022-00484-8)
Supplement: Supplementary file 1 — Supplementary Material 1 [file 13031_2022_484_MOESM1_ESM.docx]

**Supplementary data**

**Appendix 1**

**SUPPLEMENTARY APPENDIX 1: PAPERS INCLUDED IN THE REVIEW (LISTED AS CITED IN THE FINDING and DISCUSSION SEGMETNS)**

1. Banda Chitsamatanga B, Malinga W. ‘A tale of two paradoxes in response to COVID-19’: Public health system and socio-economic implications of the pandemic in South Africa and Zimbabwe. Serpa S, editor. Cogent Social Sciences. 2021 Jan 1;7(1):1869368.

2. Mayhew SH, Balabanova D, Vandi A, Mokuwa GA, Hanson T, Parker M, et al. (Re)arranging “systems of care” in the early Ebola response in Sierra Leone: An interdisciplinary analysis. Social Science & Medicine. 2021 Jul;114209.

3. Munodawafa D, Moeti MR, Phori PM, Fawcett SB, Hassaballa I, Sepers C, et al. Monitoring and Evaluating the Ebola Response Effort in Two Liberian Communities. J Community Health. 2018 Apr;43(2):321–7.

4. Odhiambo J, Jeffery C, Lako R, Devkota B, Valadez JJ. Measuring health system resilience in a highly fragile nation during protracted conflict: South Sudan 2011–15. Health Policy and Planning. 2020 Apr 1;35(3):313–22.

5. Wai KS, Khine WYK, Lim JM, Neo PHM, Tan RKJ, Ong SE. Malaysia, Myanmar and Singapore: common threads, divergences, and lessons learned in responding to the COVID-19 pandemic. The Round Table. 2021 Jan 2;110(1):84–98.

6. Amsalu R, Schulte-Hillen C, Garcia DM, Lafferty N, Morris CN, Gee S, et al. Lessons Learned From Helping Babies Survive in Humanitarian Settings. Pediatrics. 2020 Oct 1;146(Supplement_2):S208–17.

7. Denhard L, Kaviany P, Chicumbe S, Muianga C, Laisse G, Aune K, et al. How prepared is Mozambique to treat COVID-19 patients? A new approach for estimating oxygen service availability, oxygen treatment capacity, and population access to oxygen-ready treatment facilities. Int J Equity Health. 2021 Dec;20(1):90.

8. Aminizadeh M, Farrokhi M, Ebadi A, Masoumi G, Kolivand P, Khankeh H. Hospital Preparedness Challenges in Biological Disasters: A Qualitative Study. Disaster med public health prep. 2020 Nov 5;1–5.

9. McPake B, Witter S, Ssali S, Wurie H, Namakula J, Ssengooba F. Ebola in the context of conflict affected states and health systems: case studies of Northern Uganda and Sierra Leone. Confl Health. 2015 Dec;9(1):23.

10. Iskandar N, Rahbany T, Shokor A. Healthcare and Terrorism: The Lebanese Experience. :4.

11. Ochu CL, Akande OW, Oyebanji O, Aderinola O, Ogunbode O, Atteh R, et al. ‘Fighting a Global War Using a Local Strategy’: contextualism in COVID-19 response in Africa. BMJ Innov. 2021 Apr;7(2):347–55.

12. Olusola A, Olusola B, Onafeso O, Ajiola F, Adelabu S. Early geography of the coronavirus disease outbreak in Nigeria. GeoJournal [Internet]. 2020 Aug 13 [cited 2021 Dec 13]; Available from: https://link.springer.com/10.1007/s10708-020-10278-1

13. Dureab F, Al-Sakkaf M, Ismail O, Kuunibe N, Krisam J, Müller O, et al. Diphtheria outbreak in Yemen: the impact of conflict on a fragile health system. Confl Health. 2019 Dec;13(1):19.

14. Durski KN, Singaravelu S, Naidoo D, Djingarey MH, Fall IS, Yahaya AA, et al. Design thinking during a health emergency: building a national data collection and reporting system. BMC Public Health. 2020 Dec;20(1):1896.

15. McKenzie A, Abdulwahab A, Sokpo E, Mecaskey JW. Creating the Foundation for Health System Resilience in Northern Nigeria. Health Systems & Reform. 2016 Oct;2(4):357–66.

16. Ayobami O, Mark G, Kadri-Alabi Z, Achi CR, Jacob JC. COVID-19: an opportunity to re-evaluate the implementation of a One Health approach to tackling emerging infections in Nigeria and other sub-Saharan African countries. J Egypt Public Health Assoc. 2021 Dec;96(1):26.

17. Bizri NA, Alam W, Mobayed T, Tamim H, Makki M, Mushrrafieh U. COVID-19 in conflict region: the arab levant response. BMC Public Health. 2021 Dec;21(1):1590.

18. Camara S, Delamou A, Millimouno TM, Kourouma K, Ndiaye B, Thiam S. Community response to the Ebola outbreak: Contribution of community-based organisations and community leaders in four health districts in Guinea. Global Public Health. 2020 Dec 1;15(12):1767–77.

19. Barker KM, Ling EJ, Fallah M, VanDeBogert B, Kodl Y, Macauley RJ, et al. Community engagement for health system resilience: evidence from Liberia’s Ebola epidemic. Health Policy and Planning. 2020 May 1;35(4):416–23.

20. Pouraghaei M, Jannati A, Moharamzadeh P, Ghaffarzad A, Far MH, Babaie J. Challenges of Hospital Response to the Twin Earthquakes of August 21, 2012, in East Azerbaijan, Iran. Disaster med public health prep. 2017 Aug;11(4):422–30.

21. Ling EJ, Larson E, Macauley RJ, Kodl Y, VanDeBogert B, Baawo S, et al. Beyond the crisis: did the Ebola epidemic improve resilience of Liberia’s health system? Health Policy and Planning. 2017 Nov 1;32(suppl_3):iii40–7.

22. Daw MA, El-Bouzedi A, Dau AA. The assessment of efficiency and coordination within the Libyan health care system during the armed conflict-2011. Clinical Epidemiology and Global Health. 2016 Sep;4(3):120–7.

23. Haider N, Osman AY, Gadzekpo A, Akipede GO, Asogun D, Ansumana R, et al. Lockdown measures in response to COVID-19 in nine sub-Saharan African countries. BMJ Glob Health. 2020 Oct;5(10):e003319.

24. Duclos D, Ekzayez A, Ghaddar F, Checchi F, Blanchet K. Localisation and cross-border assistance to deliver humanitarian health services in North-West Syria: a qualitative inquiry for The Lancet-AUB Commission on Syria. Confl Health. 2019 Dec;13(1):20.

25. Farazmand A, Danaeefard H. Iranian Government’s Responses to the Coronavirus Pandemic (COVID-19): An Empirical Analysis. International Journal of Public Administration. 2021 Sep 10;44(11–12):931–42.

26. Khalid AF, Lavis JN, El-Jardali F, Vanstone M. The governmental health policy-development process for Syrian refugees: an embedded qualitative case studies in Lebanon and Ontario. Confl Health. 2019 Dec;13(1):48.

27. Kodish SR, Simen-Kapeu A, Beauliere J-M, Ngnie-Teta I, Jalloh MB, Pyne-Bailey S, et al. Consensus building around nutrition lessons from the 2014–16 Ebola virus disease outbreak in Guinea and Sierra Leone. Health Policy and Planning. 2019 Mar 1;34(2):83–91.

28. Nabyonga-Orem J, Gebrikidane M, Mwisongo A. Assessing policy dialogues and the role of context: Liberian case study before and during the Ebola outbreak. BMC Health Serv Res. 2016 Jul;16(S4):219.

29. McMahon SA, Ho LS, Scott K, Brown H, Miller L, Ratnayake R, et al. “We and the nurses are now working with one voice”: How community leaders and health committee members describe their role in Sierra Leone’s Ebola response. BMC Health Serv Res. 2017 Dec;17(1):495.

30. Alonge O, Sonkarlay S, Gwaikolo W, Fahim C, Cooper JL, Peters DH. Understanding the role of community resilience in addressing the Ebola virus disease epidemic in Liberia: a qualitative study (community resilience in Liberia). Global Health Action. 2019 Jan 1;12(1):1662682.

31. Richards P, Mokuwa GA, Vandi A, Mayhew SH, Ebola Gbalo Research Team. Re-analysing Ebola spread in Sierra Leone: The importance of local social dynamics. Hodges MH, editor. PLoS ONE. 2020 Nov 5;15(11):e0234823.

32. Miller NP, Milsom P, Johnson G, Bedford J, Kapeu AS, Diallo AO, et al. Community health workers during the Ebola outbreak in Guinea, Liberia, and Sierra Leone. 2018;8(2):17.

33. Koenig SP, Rouzier V, Vilbrun SC, Morose W, Collins SE, Joseph P, et al. Tuberculosis in the aftermath of the 2010 earthquake in Haiti. Bull World Health Organ. 2015 Jul 1;93(7):498–502.

34. Tappis H, Elaraby S, Elnakib S, AlShawafi NAA, BaSaleem H, Al-Gawfi IAS, et al. Reproductive, maternal, newborn and child health service delivery during conflict in Yemen: a case study. Confl Health. 2020 Dec;14(1):30.

35. Rude JM, Kortimai L, Mosoka F, Baller A, Nuha M, Katawera V, et al. Rapid response to meningococcal disease cluster in Foya district, Lofa County, Liberia January to February 2018. Pan Afr Med J [Internet]. 2019 [cited 2021 Dec 13];33. Available from: http://www.panafrican-med-journal.com/content/series/33/2/6/full/

36. Li Z-J, Tu W-X, Wang X-C, Shi G-Q, Yin Z-D, Su H-J, et al. A practical community-based response strategy to interrupt Ebola transmission in sierra Leone, 2014–2015. Infect Dis Poverty. 2016 Dec;5(1):74.

37. Kouadio K, Okeibunor J, Nsubuga P, Mihigo R, Mkanda P. Polio infrastructure strengthened disease outbreak preparedness and response in the WHO African Region. Vaccine. 2016 Oct;34(43):5175–80.

38. Bar-On E, Blumberg N, Joshi A, Gam A, Peyser A, Lee E, et al. Orthopedic Activity in Field Hospitals Following Earthquakes in Nepal and Haiti: Variability in Injuries Encountered and Collaboration with Local Available Resources Drive Optimal Response. World J Surg. 2016 Sep;40(9):2117–22.

39. Pajevic I, Hasanović M, Avdibegović E, Džubur-Kulenović A, Burgić-Radmanović M, Babić D, et al. Organization of mental healthcare in Bosnia and Herzegovina during coronavirus disease 2019 pandemic. Indian J Psychiatry. 2020;62(9):479.

40. Hierink F, Rodrigues N, Muñiz M, Panciera R, Ray N. Modelling geographical accessibility to support disaster response and rehabilitation of a healthcare system: an impact analysis of Cyclones Idai and Kenneth in Mozambique. BMJ Open. 2020 Nov;10(11):e039138.

41. Sodi T, Modipane M, Oppong Asante K, Quarshie EN-B, Asatsa S, Mutambara J, et al. Mental health policy and system preparedness to respond to COVID-19 and other health emergencies: a case study of four African countries. South African Journal of Psychology. 2021 Jun;51(2):279–92.

42. Hung YW, Law MR, Cheng L, Abramowitz S, Alcayna-Stevens L, Lurton G, et al. Impact of a free care policy on the utilisation of health services during an Ebola outbreak in the Democratic Republic of Congo: an interrupted time-series analysis. BMJ Glob Health. 2020 Jul;5(7):e002119.

43. McQuilkin PA, Udhayashankar K, Niescierenko M, Maranda L. Health-Care Access during the Ebola Virus Epidemic in Liberia. The American Journal of Tropical Medicine and Hygiene. 2017 Sep 7;97(3):931–6.

44. Plucinski MM, Guilavogui T, Sidikiba S, Diakité N, Diakité S, Dioubaté M, et al. Effect of the Ebola-virus-disease epidemic on malaria case management in Guinea, 2014: a cross-sectional survey of health facilities. The Lancet Infectious Diseases. 2015 Sep;15(9):1017–23.

45. Shapira G, Ahmed T, Drouard SHP, Amor Fernandez P, Kandpal E, Nzelu C, et al. Disruptions in maternal and child health service utilization during COVID-19: analysis from eight sub-Saharan African countries. Health Policy and Planning. 2021 Aug 12;36(7):1140–51.

46. Bompangue D, Moore S, Taty N, Impouma B, Sudre B, Manda R, et al. Description of the targeted water supply and hygiene response strategy implemented during the cholera outbreak of 2017–2018 in Kinshasa, DRC. BMC Infect Dis. 2020 Dec;20(1):226.

47. Umoke PCI, Umoke M, Eyo N, Ugwu MBBS A, Okeke E, Nwalieji CA, et al. Delay in health‐seeking behaviour: Implication to yellow fever outcome in the 2019 outbreak in Nigeria. Health Soc Care Community. 2021 May;29(3):703–11.

48. das Neves Martins Pires PH, Macaringue C, Abdirazak A, Mucufo JR, Mupueleque MA, Zakus D, et al. Covid-19 pandemic impact on maternal and child health services access in Nampula, Mozambique: a mixed methods research. BMC Health Serv Res. 2021 Dec;21(1):860.

49. Hassanain SA, Edwards JK, Venables E, Ali E, Adam K, Hussien H, et al. Conflict and tuberculosis in Sudan: a 10-year review of the National Tuberculosis Programme, 2004-2014. Confl Health. 2018 Dec;12(1):18.

50. Abramowitz SA, McLean KE, McKune SL, Bardosh KL, Fallah M, Monger J, et al. Community-Centered Responses to Ebola in Urban Liberia: The View from Below. Bausch DG, editor. PLoS Negl Trop Dis. 2015 Apr 9;9(4):e0003706.

51. McLean KE, Abramowitz SA, Ball JD, Monger J, Tehoungue K, McKune SL, et al. Community-based reports of morbidity, mortality, and health-seeking behaviours in four Monrovia communities during the West African Ebola epidemic. Global Public Health. 2018 May 4;13(5):528–44.

52. Malembaka EB, Karemere H, Bisimwa Balaluka G, Altare C, Odikro MA, Lwamushi SM, et al. Are people most in need utilising health facilities in post-conflict settings? A cross-sectional study from South Kivu, eastern DR Congo. Global Health Action. 2020 Dec 31;13(1):1740419.

53. Kolie D, Van De Pas R, Delamou A, Dioubaté N, Beavogui FT, Bouedouno P, et al. Retention of healthcare workers 1 year after recruitment and deployment in rural settings: an experience post-Ebola in five health districts in Guinea. Hum Resour Health. 2021 Dec;19(1):67.

54. Witter S, Wurie H, Chandiwana P, Namakula J, So S, Alonso-Garbayo A, et al. How do health workers experience and cope with shocks? Learning from four fragile and conflict-affected health systems in Uganda, Sierra Leone, Zimbabwe and Cambodia. Health Policy and Planning. 2017 Nov 1;32(suppl_3):iii3–13.

55. Fardousi N, Douedari Y, Howard N. Healthcare under siege: a qualitative study of health-worker responses to targeting and besiegement in Syria. BMJ Open. 2019 Sep;9(9):e029651.

56. Raven J, Wurie H, Witter S. Health workers’ experiences of coping with the Ebola epidemic in Sierra Leone’s health system: a qualitative study. BMC Health Serv Res. 2018 Dec;18(1):251.

57. Poortaghi S, Shahmari M, Ghobadi A. Exploring nursing managers’ perceptions of nursing workforce management during the outbreak of COVID-19: a content analysis study. BMC Nurs. 2021 Dec;20(1):27.

58. Turke S, Nehrling S, Adebayo SO, Akilimali P, Idiodi I, Mwangi A, et al. Remote Interviewer Training for COVID-19 Data Collection: Challenges and Lessons Learned From 3 Countries in Sub-Saharan Africa. Glob Health Sci Pract. 2021 Mar 31;9(1):177–86.

59. Garber K, Fox C, Abdalla M, Tatem A, Qirbi N, Lloyd-Braff L, et al. Estimating access to health care in Yemen, a complex humanitarian emergency setting: a descriptive applied geospatial analysis. The Lancet Global Health. 2020 Nov;8(11):e1435–43.

60. Durski KN, Singaravelu S, Teo J, Naidoo D, Bawo L, Jambai A, et al. Development, Use, and Impact of a Global Laboratory Database During the 2014 Ebola Outbreak in West Africa. The Journal of Infectious Diseases. 2017 Jun 15;215(12):1799–806.
